# Targeting solid tumors with TCR-T cells: mechanisms, progress, and challenges

**DOI:** 10.3389/fonc.2026.1810903

**Published:** 2026-05-07

**Authors:** Wenfang Hu, Zhongyu Zhang, Mengyao Pan, Weiye Wang, Zibing Wang

**Affiliations:** 1 Department of Immunotherapy, Affiliated Cancer Hospital of Zhengzhou University & Henan Cancer Hospital, Zhengzhou, China; 2 Department of Medical Oncology, The Second Affiliated Hospital of Zhengzhou University, Zhengzhou, China; 3 Department of Neurology, Cangzhou People’s Hospital, Cangzhou, China

**Keywords:** adoptive cell therapy, antigen presentation, peptide–HLA, solid tumors, TCR-T therapy, tumor microenvironment

## Abstract

T-cell receptor-engineered T-cell (TCR-T) therapy has emerged as a promising strategy for solid tumors because it enables recognition of intracellular antigens presented by human leukocyte antigen (HLA) molecules, thereby extending targetability beyond cell-surface proteins. However, its clinical activity remains inconsistent because of HLA restriction, heterogeneous antigen expression, unstable antigen presentation, and an immunosuppressive tumor microenvironment. In this review, we summarize the biological basis of TCR-T therapy in solid tumors, including peptide–HLA recognition, target selection, antigen-presentation barriers, and mechanisms of tumor-cell killing. We then review current clinical progress across major solid tumor types, highlighting meaningful responses in selected biomarker-defined settings while noting that efficacy in many epithelial cancers remains limited. Current evidence further indicates that target recognition alone is insufficient for durable tumor control; sustained benefit also depends on preserved antigen presentation, effective tumor trafficking, resistance to suppressive signals, and maintenance of T-cell fitness. We also discuss emerging strategies to improve therapeutic performance, including precision receptor engineering, multi-HLA target development, microenvironment-focused armoring, and manufacturing optimization. Overall, TCR-T therapy provides a compelling framework for solid-tumor treatment, but broader and more durable benefit will require integrated advances in target selection, safety design, and cellular engineering.

## 1 Introduction

Solid tumors remain challenging targets for adoptive T-cell therapy because their actionable antigens are heterogeneous, antigen presentation is often unstable, and the tumor microenvironment (TME) actively restricts T-cell trafficking and effector function (1, 2). Although chemotherapy and radiotherapy continue to improve clinical outcomes in many settings, their therapeutic index is often limited by incomplete tumor specificity and cumulative toxicities (3). Genetically engineered T-cell therapies, particularly chimeric antigen receptor T cells (CAR-T), have shown transformative efficacy in selected hematologic malignancies, but their application in solid tumors has produced more variable outcomes (4, 5). Against this backdrop, TCR-T therapy offers a distinct advantage by recognizing intracellular antigens presented by human leukocyte antigen (HLA) molecules, thereby extending targetability beyond cell-surface proteins (6). However, this approach remains inherently constrained by HLA restriction and by the need to preserve antitumor potency while minimizing on-target/off-tumor toxicity (7). In this review, we summarize the biological rationale, emerging clinical progress, and major translational challenges of TCR-T therapy in solid tumors, with a focus on factors that shape its therapeutic potential and current limitations.

## 2 Biological basis of TCR-T therapy

The therapeutic activity of TCR-T cells in solid tumors is determined by several interrelated biological factors, including receptor architecture, target class, HLA restriction, antigen-presentation competence, and the capacity to sustain effector function. This section summarizes the mechanistic principles most relevant to subsequent clinical interpretation, with a focus on peptide–HLA recognition, target selection, antigen-presentation barriers, and tumor cell killing. Together, these principles provide a biological framework for understanding the heterogeneous clinical activity of TCR-T therapy across solid tumors.

### 2.1 Molecular architecture and signaling initiation of the TCR complex

To contextualize antigen selection and receptor design in TCR-T therapy, it is useful to briefly outline the structural features that govern peptide–HLA recognition and signal initiation. Current therapeutic TCR-T platforms are predominantly based on αβ T-cell receptors (αβ TCRs), in which antigen specificity is encoded by the variable domains (6, 8). In these receptors, complementarity-determining regions 1 and 2 (CDR1 and CDR2) interact primarily with the HLA helices, whereas CDR3 usually contributes most of the peptide-contacting interactions that determine fine specificity (9). Productive activation requires assembly of the TCR with the CD3 signaling complex, which consists of CD3γϵ, CD3δϵ, and ζζ dimers (10). This assembly is stabilized by charged residues within the transmembrane regions and transmits downstream signals through immunoreceptor tyrosine-based activation motifs (ITAMs) (11).

By contrast, γδ T cells represent a smaller and more heterogeneous T-cell compartment and can recognize non-peptidic or non-classical ligands with less reliance on classical HLA restriction (12, 13). Although this biology is increasingly being explored in solid-tumor immunotherapy, current clinical TCR-T development remains focused primarily on αβ TCRs (8).

This signaling architecture also distinguishes TCR-T cells from CAR-T cells. CAR-T cells use synthetic receptors with built-in signaling domains to recognize surface antigens independently of HLA, whereas TCR-T cells rely on native TCR–CD3 signaling triggered by peptide–HLA recognition (14, 15). Accordingly, receptor specificity, HLA context, and the integrity of antigen presentation are fundamental determinants of both efficacy and safety in TCR-T therapy (8).

### 2.2 Peptide targets in solid tumors

Against this biological backdrop, peptide targets in solid tumors are commonly classified as tumor-associated antigens (TAAs) or tumor-specific antigens (TSAs). This distinction remains conceptually useful, but it is most informative when interpreted within a clinically relevant framework. TAAs are self-derived antigens that are aberrantly expressed, overexpressed, retained, or lineage-restricted in malignant tissues. A major subgroup is formed by cancer-testis antigens (CTAs), exemplified by NY-ESO-1/CTAG1B and members of the MAGE family. Other TAA categories include differentiation antigens that remain expressed in malignant tissues, particularly melanocytic lineage antigens such as MART-1, gp100/PMEL, and tyrosinase in melanoma. Clinical experience with this antigen class has highlighted the risk of on-target/off-tumor injury in normal tissues that share the same lineage program (16–18).

By contrast, TSAs are antigens that are absent from, or only minimally represented in, normal tissues and may therefore offer a more favorable tumor-specificity profile. Representative TSA classes include viral antigens in virus-associated cancers, such as HPV E6/E7 (19). HBV-derived epitopes represent a parallel example in HBV-associated hepatocellular carcinoma (20). Neoantigen targets may also be recurrent and shared across patients, as illustrated by hotspot mutations such as KRAS (21). Mutant TP53 provides another example of a potentially shared neoantigen class with broader relevance across epithelial cancers (22). By contrast, other neoantigen targets are non-recurrent and patient-specific, as exemplified by personalized neoantigen-based approaches that rely on individualized tumor sequencing, epitope selection, and TCR engineering workflows (23). In selected disease settings, endogenous retroviral products may also function as TSA-like targets. A representative example is human endogenous retrovirus E (HERV-E), which is selectively expressed in clear cell renal cell carcinoma and can generate immunogenic peptides recognizable by T cells (24).

Because TCR recognition depends on both intracellular peptide processing and stable peptide–HLA presentation, target class alone does not determine clinical tractability or therapeutic suitability. Both TAA- and TSA-directed strategies remain constrained by HLA restriction. Shared TAAs may support broader clinical deployability, but they require stringent mitigation of on-target/off-tumor toxicity (16, 18). TSAs often offer greater tumor selectivity, yet their practical use may be constrained by mutation prevalence, biomarker-defined eligibility, and, in personalized settings, the time and complexity of individualized manufacturing (6, 23). Loss or instability of peptide–HLA presentation can undermine either class and should not be framed as a TSA-specific liability (2). Accordingly, target selection in TCR-T therapy should be evaluated across four interrelated dimensions: tumor specificity, target prevalence, HLA context, and the likelihood of durable and homogeneous antigen presentation.

### 2.3 HLA diversity and antigen-presentation barriers in TCR-T therapy

The clinical applicability of TCR-T therapy is fundamentally constrained by the extensive polymorphism of HLA class I molecules (25). Because therapeutic αβ TCRs recognize their cognate peptides only in the context of specific HLA allotypes, HLA restriction functions as a primary eligibility filter from the earliest stages of clinical development (26). This is not merely an operational detail, but a factor that directly influences screening yield, trial accrual, market reach, and the feasibility of extending a given target beyond a narrowly defined biomarker-selected population (27).

Population-level carrier frequencies provide a more intuitive explanation for why certain HLA backbones have dominated early TCR-T development (25). Large donor-registry analyses further show that HLA composition differs substantially across ancestry groups and directly affects matchability at scale (28). HLA-A*02:01 has served as a major clinical backbone in part because a substantial fraction of patients in Western populations carry this allele, whereas carriage is markedly less common in Asian or Pacific Islander populations (27). Likewise, HLA-A*11:01 is carried by a substantial proportion of individuals in several Asian populations and has therefore emerged as an increasingly important parallel development axis in Asian-enriched programs (29, 30). These population differences are clinically consequential because they directly affect screening yield, trial accrual, and the real-world deployability of HLA-restricted TCR-T products.

However, HLA matching addresses only part of the problem (31). Even when patients carry the relevant HLA allele, tumor recognition may still fail because antigen presentation is impaired at the tumor level (2). Allele-specific HLA loss is one such mechanism and was detected in 40% of non-small-cell lung cancers in a landmark analysis (32). Acquired mutations affecting β2-microglobulin can similarly disrupt antigen presentation and contribute to resistance under T-cell-based immune pressure (33). Defects in interferon-γ signaling, including JAK1 or JAK2 loss, can generate tumor lesions that are HLA class I-negative and resistant to T-cell attack (34). More broadly, defects in antigen-processing machinery (APM) components are frequently observed across cancers and are clinically relevant to T-cell-based immunotherapy (31). Collectively, these abnormalities can reduce or abolish surface peptide–HLA complexes despite nominal target positivity, thereby contributing to screening attrition, diminished responses, and lesion-to-lesion heterogeneity in clinical activity (2).

For this reason, HLA typing alone should not be considered sufficient for biomarker selection. A more clinically useful framework should integrate HLA genotyping with tumor-level assessment of target expression and antigen-presentation competence (35). Accordingly, the most realistic strategy for expanding patient access is not to imply that peptide–HLA restriction can be bypassed, but rather to develop parallel TCR portfolios across multiple prevalent HLA allotypes (27). In parallel, more rigorous assessment of HLA/APM integrity should be incorporated into biomarker triage for patient selection (31). In this context, broader HLA coverage and more rigorous evaluation of antigen-presentation competence should be viewed as complementary, rather than competing, requirements for the clinical translation of TCR-T therapy.

### 2.4 Tumor killing mechanisms of TCR-T cells

Tumor killing by TCR-T cells depends on successful recognition of cognate peptide–HLA complexes on the target-cell surface (6). Unlike CAR-based formats, which bind surface molecules directly, TCR-T cells initiate cytotoxicity only after recognizing intracellular antigen-derived peptides presented by HLA molecules (36). The efficiency of this process is determined by several interacting variables, including the abundance and stability of peptide–HLA complexes, the biophysical properties of the engineered TCR–peptide–HLA (pHLA) interaction, and the integrity of antigen-processing and presentation pathways (35). In solid tumors, heterogeneous target display and variable tumor susceptibility may restrict efficient killing to only a subset of tumor cells, thereby contributing to incomplete responses and immune escape (37).

Following peptide–HLA engagement, the engineered αβ TCR signals through the native CD3 complex and initiates canonical ITAM-dependent signaling cascades that culminate in the activation of NFAT, NF-κB, and AP-1 transcriptional programs (38, 39). Although this signaling architecture is shared with endogenous T cells, the magnitude and duration of signaling in TCR-T products may vary according to receptor affinity and surface density, and may also be influenced by how the transgenic receptor is introduced and regulated within the cell (40). Affinity and potency engineering may improve sensitivity to low-density peptide–HLA complexes, but these gains must be interpreted alongside specificity and safety, because such modifications can reshape cross-reactivity profiles (41).

In solid tumors, sustained killing is often constrained by insufficient supportive signaling within the TME. TCR triggering may occur under conditions of limited co-stimulatory support, repeated antigen stimulation, and abundant inhibitory or suppressive cues. Under these conditions, durable effector function is difficult to maintain. Consistent with this view, recent preclinical work showed that T-cell-restricted IL-15 and IL-21 armoring enhanced the resilience and antitumor efficacy of both CAR- and TCR-engineered T cells and reduced dysfunction after repeated stimulation in solid-tumor models (42).

Once a productive cytotoxic synapse has formed, tumor elimination commonly proceeds through granule-mediated cytotoxicity delivered across repeated encounters and, in some settings, through Fas-dependent death-receptor signaling (43). In solid tumors, effective killing may require cumulative sublethal damage inflicted through multiple T-cell contacts rather than a single efficient hit (44). At the same time, tumor-derived metabolites can impair immunological synapse formation, intratumoral hypoxia can drive ATF4-dependent CD8^+^ T-cell dysfunction, and tumor-intrinsic glycolysis can reduce sensitivity to TNF-mediated bystander killing (45). In this context, functional exhaustion is better understood as a progressive failure to sustain repeated cytotoxic interactions under suppressive conditions than as a fixed terminal state (46, 47).

Taken together, effective TCR-T-mediated tumor control requires the convergence of three conditions: a sufficiently abundant and stable peptide–HLA target, an engineered receptor with an appropriate balance between potency and specificity, and a cellular state capable of maintaining effector function within the TME (46). These principles provide a mechanistic framework for interpreting the heterogeneous clinical activity observed across solid tumors.

## 3 Clinical applications and advances

TCR-T therapy has attracted increasing interest in solid tumors because it enables HLA-restricted recognition of intracellular antigens that are inaccessible to conventional surface-directed approaches (48, 49). To date, the clearest clinical activity has been observed in synovial sarcoma and melanoma, where biomarker-selected trials targeting CTAs have produced objective responses, although the magnitude and durability of benefit have varied across products and cohorts (50, 51). In several epithelial malignancies, early clinical studies have likewise established feasibility and generally manageable safety profiles; however, antitumor activity has been less consistent and clinical development remains less mature (49). The following sections summarize the available evidence by tumor type, with an emphasis on target selection, clinical-trial evidence, efficacy, safety, and the disease-specific context most relevant to therapeutic benefit.

### 3.1 Sarcoma

Among solid tumors, sarcoma currently represents the most clinically mature setting for TCR-T therapy, largely because actionable CTAs are relatively well characterized in selected subtypes and the supporting clinical evidence is stronger than that in most other solid tumors. Early proof of concept came from NY-ESO-1-directed engineered T-cell therapy, which induced objective regressions in metastatic synovial sarcoma and demonstrated that biomarker-selected sarcomas could be clinically responsive to TCR redirection (52). These early findings established sarcoma not only as a feasible disease context for TCR-T therapy, but also as a paradigm in which biomarker-guided antigen selection and HLA restriction could be translated into clinically meaningful activity.

More recent progress has been driven by MAGE-A4-directed therapy. In the phase 1 trial of afamitresgene autoleucel (afami-cel), responses were enriched in synovial sarcoma, with 7 responses among 16 patients in that subgroup and a median duration of response of 28.1 weeks (48). These observations were subsequently reinforced by the phase 2 SPEARHEAD-1 study, which demonstrated durable activity in heavily pretreated patients with HLA-A*02-positive, MAGE-A4-expressing synovial sarcoma. In this cohort, the objective response rate was 39% (17/44). Cytokine release syndrome (CRS) was common overall but was usually low grade, with only one grade 3 event reported, whereas grade 3 or higher toxicities were dominated by expected post-lymphodepletion cytopenias (53). Based on these data, afami-cel received U.S. Food and Drug Administration (FDA) accelerated approval on August 2, 2024, for adults with unresectable or metastatic synovial sarcoma after prior chemotherapy whose tumors express MAGE-A4 and who are positive for HLA-A*02:01P, HLA-A*02:02P, HLA-A*02:03P, or HLA-A*02:06P, thereby becoming the first FDA-approved engineered TCR product for a solid tumor (54). Importantly, this indication does not include HLA-A*02:05P (55).

A second major line of progress has come from NY-ESO-1/LAGE-1a-directed therapy. Importantly, NY-ESO-1 and LAGE-1a are distinct cancer-testis antigen genes, but they are highly homologous and share a therapeutically relevant HLA-A*02:01-presented epitope. For this reason, they are often developed and discussed together in engineered T-cell programs. After the initial National Institutes of Health (NIH) experience established proof of concept in synovial sarcoma (52), the subsequent development of letetresgene autoleucel (lete-cel) expanded this signal into a larger sarcoma-focused program. In the phase 2 IGNYTE-ESO analysis, 27 of 64 patients with synovial sarcoma or myxoid/round-cell liposarcoma achieved responses defined by the Response Evaluation Criteria in Solid Tumors (RECIST) on independent review, corresponding to an objective response rate of 42%. Subgroup response rates were 41% in synovial sarcoma and 43% in myxoid/round-cell liposarcoma (MRCLS), with a median duration of response of 12.2 months overall and 18.3 months in synovial sarcoma (56, 57). These findings supported continued registration-oriented development, and in January 2025 the FDA granted Breakthrough Therapy Designation to lete-cel for unresectable or metastatic MRCLS after prior anthracycline-based chemotherapy in patients positive for HLA-A*02:01, HLA-A*02:05, or HLA-A*02:06 whose tumors express NY-ESO-1 (57).

Other engineered TCR programs are also under investigation, but their roles in sarcoma remain less mature than those of the leading CTA-directed products. Although MAGE-A4- and NY-ESO-1/LAGE-1a-directed programs have already generated registration-relevant efficacy signals in biomarker-selected sarcoma, comparable sarcoma-specific peer-reviewed evidence for newer targets remains limited, and subtype-specific interpretation remains essential (53, 56). This is particularly important when interpreting mixed solid-tumor studies, in which encouraging signals should not be extrapolated to sarcoma without dedicated sarcoma-specific readouts.

Taken together, sarcoma currently provides the strongest clinical proof of concept for TCR-T therapy in solid tumors. The field is now defined not only by early feasibility studies targeting NY-ESO-1, but also by a more mature therapeutic landscape in which afami-cel has achieved regulatory approval and lete-cel has advanced into late-stage registration-oriented development in selected sarcoma subtypes (54).

### 3.2 Melanoma

Melanoma remains one of the most informative disease settings for TCR-T development, but its clinical history also illustrates why antigen class is a critical determinant of both efficacy and safety. Early studies targeting melanocytic differentiation antigens, particularly MART-1 and gp100, established proof of antitumor activity, yet they also revealed a narrow therapeutic window because engineered T cells damaged normal melanocytes in the skin, eyes, and ears (17, 18). These early experiences established melanoma not only as a proof-of-concept indication, but also as a cautionary model of on-target toxicity associated with lineage-restricted antigens.

Subsequent studies suggested that CTAs may be more clinically tractable in melanoma than melanocytic differentiation antigens. NY-ESO-1-directed TCR-T produced objective responses in melanoma, including durable complete regressions in early clinical studies and an objective response rate of 55% in the long-term follow-up of a larger cohort (50, 52). In this setting, the appeal of shared CTAs lies not in an abstract assumption of a broader therapeutic index, but in the empirical observation that they can mediate meaningful antitumor activity without reproducing the characteristic melanocyte-lineage toxicities associated with MART-1- or gp100-directed approaches (50).

Melanoma also provided one of the most consequential safety lessons in the TCR-T field. In the affinity-enhanced MAGE-A3 program, fatal cardiotoxicity was traced to off-target cross-reactivity with titin rather than to the intended tumor-restricted recognition event. This case has therefore become a defining benchmark for preclinical specificity assessment, rather than merely a historical melanoma trial (58, 59).

More recent progress in melanoma has been driven by PRAME-directed therapy. In the 2025 Nature Medicine phase 1 report of IMA203 across PRAME-positive solid tumors, the product showed promising activity, with severe CRS reported in 4.9% of treated patients and no severe neurotoxicity observed in the overall study population (49). A melanoma-focused 2025 clinical update reported encouraging activity of IMA203 in heavily pretreated metastatic melanoma, with a confirmed objective response rate of 56% and disease control in 91% of evaluable patients (51). Importantly, the safety profile was more informative than a generic statement of “manageable safety”: lymphodepletion-associated cytopenias were the dominant adverse events, CRS was mostly mild to moderate, immune effector cell-associated neurotoxicity syndrome (ICANS) was infrequent, and no grade 5 IMA203-related adverse events were reported (51). Separate phase I abstract-level data also suggested encouraging activity of IMA203 in previously treated advanced or metastatic uveal melanoma, although these findings should currently be interpreted as preliminary (60). These data have helped move PRAME from an emerging target toward a registration-oriented development pathway, with the phase 3 SUPRAME trial now enrolling patients with previously treated unresectable or metastatic cutaneous melanoma (61).

Personalized neoantigen-directed T-cell therapy has provided a distinct, although still preliminary, signal of progress. In the 2025 first-in-human BNT221 study, personalized neoantigen-specific products were successfully manufactured for all enrolled patients, neoantigen-specific clonotypes were detected in both blood and tumor after infusion, and treatment was well tolerated without CRS, ICANS, or macrophage activation syndrome. However, the antitumor signal remained modest: six of nine patients achieved stable disease as their best overall response, with tumor reductions of 20% or less in four of those patients (62). These findings suggest that melanoma now exemplifies two parallel developmental trajectories in TCR-T therapy: a more advanced shared-antigen pathway led by PRAME, and a personalized neoantigen pathway that has clearly demonstrated feasibility but still faces major bottlenecks in target prioritization, manufacturing speed, and depth of clinical response.

Importantly, personalized TCR-T development is no longer confined to melanoma. A clinical-grade non-viral precision TCR replacement platform has demonstrated the feasibility of isolating, manufacturing, and infusing patient-specific neoantigen-reactive TCR products across refractory solid tumors (23). In parallel, adoptive transfer of autologous lymphocytes transduced with personalized neoantigen-reactive TCRs has shown early evidence of tumor regression in metastatic colorectal cancer (63). More recently, biopsy-compatible discovery platforms have further strengthened the translational basis for individualized TCR-T development (64). Together, these studies suggest that personalized neoantigen-directed TCR therapy is evolving from a melanoma-centered proof of concept into a broader platform strategy across solid tumors.

Overall, melanoma remains one of the clearest disease frameworks for understanding both the opportunities and liabilities of TCR-T therapy in solid tumors. Current momentum is driven less by historical MART-1 or gp100 programs than by PRAME-based shared-antigen platforms that have entered phase 3 testing, as well as by emerging high-throughput TCR discovery approaches that may broaden individualized neoantigen targeting once current translational bottlenecks are addressed (65, 66).

### 3.3 Non–small cell lung cancer

In non–small cell lung cancer (NSCLC), TCR-T therapy remains at an early but increasingly informative stage of clinical development. Early clinical experience centered on NY-ESO-1/LAGE-1a-directed therapy and highlighted the practical constraints imposed by HLA restriction and limited antigen prevalence. As noted above, NY-ESO-1 and LAGE-1a are distinct but highly homologous genes that share a therapeutically relevant HLA-A*02:01-restricted epitope. This explains why they are commonly discussed together in clinical TCR-T development. In the pilot lete-cel studies reported by Altan et al., more than 2,500 patients were screened, yet only 18 ultimately received treatment, underscoring how biomarker selection can markedly narrow the treatable population (67). Despite this highly selective screening process, the studies established clinical feasibility and a manageable safety profile. Cytopenias and CRS were the most common treatment-emergent adverse events, no fatal treatment-related serious adverse events were reported, and the addition of pembrolizumab did not appear to increase toxicity relative to lete-cel alone. Antitumor activity, however, was limited, with only 1 of 18 treated patients achieving a durable partial response that persisted for 18 months, whereas most others experienced non-durable disease control or progression (67).

More recent progress in NSCLC has been driven by MAGE-A4-directed therapy. In the ongoing phase 1 SURPASS trial of ADP-A2M4CD8, clinical activity has been observed across multiple MAGE-A4-positive solid tumors (68). In the 2023 update, objective responses were reported in 16 of 46 evaluable patients overall. In the NSCLC subset, 1 partial response was observed among 9 patients, and 2 additional patients achieved stable disease; the median duration of response across the overall cohort was 4.9 months. Severe CRS was uncommon, with grade 3 or higher CRS reported in 6% of patients in that update (68). A subsequent 2023 translational analysis further supported a favorable benefit-to-risk profile for ADP-A2M4CD8 across the broader solid-tumor population, although NSCLC-specific efficacy remained modest and patient numbers were still limited. Collectively, these findings indicate that MAGE-A4-directed TCR-T can induce objective responses in selected NSCLC cases, although durable clinical benefit in this disease has yet to be established (68).

Beyond NY-ESO-1/LAGE-1a and MAGE-A4, additional targets are under investigation, but their relevance to NSCLC remains less mature. PRAME-directed TCR-T has shown encouraging activity in mixed solid-tumor cohorts, particularly in melanoma-enriched populations, but dedicated NSCLC-specific efficacy readouts have not yet been clearly reported in peer-reviewed form. Extrapolation to NSCLC should therefore remain cautious (49). Likewise, KK-LC-1 has attracted interest because it is expressed in subsets of epithelial cancers, including lung cancer, and a first-in-human phase 1 TCR-T trial is ongoing in metastatic KK-LC-1-positive epithelial tumors. However, mature NSCLC-specific efficacy data remain unavailable (69, 70).

Overall, current studies support the feasibility of TCR-T therapy in biomarker-selected NSCLC and confirm that objective responses are possible, but the field remains constrained by low effective screening yield, small treated cohorts, and limited durability of benefit. At present, NSCLC is best regarded as an early validation setting for epithelial-tumor TCR-T rather than as a clinically mature disease context, with NY-ESO-1/LAGE-1a- and MAGE-A4-directed programs providing the principal human proof of concept in this disease (67, 68).

### 3.4 Pancreatic ductal adenocarcinoma

Pancreatic ductal adenocarcinoma (PDAC) remains one of the most challenging solid-tumor settings for TCR-T therapy, but it has emerged as a leading epithelial model for KRAS-directed adoptive cell therapy (71). The first clear clinical proof of concept came from a 2022 New England Journal of Medicine report, in which autologous T cells engineered to express HLA-C*08:02-restricted TCRs targeting KRAS G12D induced a RECIST partial response of 72% in metastatic PDAC, with engineered T cells still accounting for more than 2% of circulating peripheral-blood T cells 6 months after infusion (21).

Recent progress has moved beyond single-patient proof of concept. In a 2026 single-arm phase 1/2 trial of HLA-A*11:01-restricted KRAS G12V-specific TCR-T in recurrent pancreatic cancer, five patients were treated. Grade 3 or higher adverse events were mainly lymphodepletion-related hematologic toxicities, one patient with liver metastases achieved a complete response lasting 5.5 months, and two patients had short-term stable disease. However, no responses were observed after repeat infusion, and two patients showed evidence of likely antibody-mediated hyperacute rejection of the engineered T cells after retreatment (72). Together, these studies indicate that mutant KRAS-directed TCR-T can mediate objective tumor regression in PDAC, while also highlighting unresolved challenges related to durability, repeat dosing, and engineered-receptor immunogenicity.

Broader shared-neoantigen development is also beginning to expand beyond isolated case reports. A National Cancer Institute (NCI) phase 1/2 study is evaluating HLA-A*11:01-restricted KRAS G12D-reactive TCR-transduced lymphocytes in advanced solid tumors, including pancreatic cancer (73). A separate first-in-human phase 1 program is testing AFNT-211, a high-avidity HLA-A*11:01-restricted KRAS G12V-specific engineered TCR platform, in advanced or metastatic solid tumors, with planned expansion in pancreatic cancer (74). In parallel, NT-112 is being evaluated in a phase 1 study as an autologous TCR-T product targeting KRAS G12D presented by HLA-C*08:02 (75), whereas NT-175 is in phase 1 testing for HLA-A*02:01-positive advanced solid tumors harboring TP53 R175H (76). AZD0240 is also being investigated in an early-phase study as an autologous TCR-T product targeting KRAS G12D in the context of HLA-A*11:01 (77). Together, these programs indicate that recurrent shared neoantigens are becoming a more systematic development axis in solid-tumor TCR-T therapy.

Beyond KRAS, mesothelin remains an exploratory target in PDAC. Preliminary conference-level safety data from a first-in-human phase I study suggested that FH-TCR-TMSLN was generally well tolerated and had not shown evidence of on-target toxicity at the time of reporting, although efficacy data remain immature (78). Another emerging direction is target discovery through immunopeptidomics. A 2025 Science study showed that noncanonical “cryptic” HLA-I peptides are abundant in the pancreatic cancer immunopeptidome, that approximately 30% display cancer-restricted translation, and that TCR-redirected T cells against selected cryptic antigens can kill patient-derived pancreatic cancer organoids. These findings remain preclinical, but they suggest that future PDAC TCR-T strategies may extend beyond canonical KRAS hotspot mutations (79).

Taken together, PDAC remains a challenging but increasingly relevant setting for TCR-T therapy. The clearest clinical evidence still comes from KRAS-directed approaches, beginning with the landmark KRAS G12D case report and extending to the first small KRAS G12V cohort study, whereas mesothelin- and cryptic-antigen-based strategies remain exploratory and have not yet established durable clinical benefit.

### 3.5 Renal cell carcinoma

In renal cell carcinoma (RCC), the clinical development of TCR-T therapy remains limited and has been concentrated primarily in clear-cell RCC (ccRCC). The most distinctive line of development has centered on HERV-E-derived antigens, whose expression appears to be highly restricted to ccRCC and mechanistically linked to loss of von Hippel–Lindau (VHL) function. This makes HERV-E one of the most tumor-selective shared targets investigated in this disease (80, 81).

The first clinical proof of concept has come from an HLA-A*11:01-restricted HERV-E-specific TCR-T program. In the phase I study reported by Nadal and colleagues, 17 patients with metastatic ccRCC were enrolled, and 15 proceeded to infusion after cyclophosphamide/fludarabine lymphodepletion and aldesleukin support (82). No dose-limiting toxicities, off-target toxicities, or treatment-related deaths were reported. The best overall response consisted of one partial response, while approximately 29% of patients achieved stable disease lasting at least 8 weeks. These findings indicate that HERV-E-directed TCR-T can be administered safely, while recent preclinical work further supports the biologic rationale for this target in ccRCC (83).

Beyond HERV-E, other shared antigens have also been considered, but RCC-specific clinical evidence remains sparse. NY-ESO-1 expression has been documented in a subset of RCC, with higher expression reported in metastatic than in primary specimens, suggesting a biological rationale for antigen-directed therapy in selected cases (84). However, the publicly available literature still does not provide a mature RCC-specific treated cohort with clearly reported TCR-T efficacy outcomes comparable to those now available for melanoma, sarcoma, or even NSCLC.

Taken together, RCC currently represents a biologically compelling but clinically early setting for TCR-T therapy. The field’s main advance has been the development of HERV-E-directed therapy in ccRCC, which provides early support for antigen selectivity and an acceptable safety profile. However, evidence for consistent and durable antitumor benefit remains limited.

### 3.6 HBV-associated hepatocellular carcinoma

HBV-associated hepatocellular carcinoma (HBV-HCC) represents a distinctive setting for TCR-T therapy because its target is virally derived rather than a conventional tumor-associated antigen. Early clinical proof of concept came from a liver-transplant recipient with metastatic HBV-HCC who was treated with autologous hepatitis B surface antigen (HBsAg)-specific TCR-redirected T cells, thereby establishing the feasibility of HBV-directed adoptive T-cell therapy in this disease (85). This setting is also unusual because antiviral pharmacodynamic effects, including declines in serum HBsAg and HBV DNA, may accompany antitumor activity, while transient transaminase elevations may reflect intended on-target cytolysis of HBV-antigen-expressing hepatocytes and tumor cells rather than classical off-target toxicity (86).

The first prospective phase I data in advanced HBV-HCC came from short-lived mRNA HBV-specific TCR-T therapy. In the 2021 dose-escalation study by Meng et al., eight patients with advanced HBV-HCC were treated. Therapy was generally well tolerated, one patient achieved a partial response lasting 27.7 months, and broader antiviral activity, including reduction or stabilization of HBsAg and HBV DNA, was observed across the cohort. A subsequent phase I study further showed that repeated infusions of mRNA-electroporated HBV-specific TCR-T cells were well tolerated in patients with recurrent HBV-HCC after liver transplantation (87).

More recent progress has been driven by SCG101, an autologous HBsAg-specific, HLA-A*02:01-restricted TCR-T product with stable receptor expression. In the investigator-initiated trial reported in Gut, six patients with advanced HBV-HCC received SCG101 after cyclophosphamide/fludarabine lymphodepletion. All developed significant but transient alanine aminotransferase (ALT) elevation within 1 week, no neurotoxicity was reported, CRS reached up to grade 3 but remained manageable, and the study suggested preliminary antiviral and antitumor activity (88). Preliminary late-breaking data presented at the European Association for the Study of the Liver (EASL) 2025 meeting extended this signal in a heavily pretreated multicenter cohort, in which 94% of evaluable patients achieved at least a 1.0 log10 decline in serum HBsAg within 28 days and 47% showed measurable tumor regression (89). In parallel, HBV-specific TCR-T development has continued through platform optimization, including a pilot study combining mRNA electroporation with lentiviral transduction in recurrent HBV-HCC after liver transplantation (20) and an ongoing phase 2 study of LioCyx-M as monotherapy or in combination with Lenvatinib (90).

Taken together, HBV-HCC remains one of the most distinctive current use cases for TCR-T therapy in solid tumors, with early evidence of both antiviral and antitumor activity. However, larger cohorts and longer follow-up are still needed to define durability and the role of combination strategies.

### 3.7 HPV-driven malignancies

HPV-driven malignancies represent one of the clearest virus-associated settings for TCR-T therapy because the viral oncoproteins E6 and E7 are constitutively expressed in tumor cells but absent from normal tissues. Early translational work established that HPV16-positive epithelial cancers could be recognized and killed by E6-specific TCR-engineered T cells, thereby providing the mechanistic foundation for subsequent clinical development (91). The first clinical proof of concept then came from HPV16 E6-directed therapy. In a first-in-human phase I/II study, 12 patients with metastatic HPV16-positive epithelial cancers were treated, no dose-limiting toxicities were observed, and 2 patients achieved objective responses (19).

A stronger clinical signal was subsequently reported with HPV16 E7-directed TCR-T therapy. In the first-in-human phase I study by Nagarsheth and colleagues, objective responses were observed in 6 of 12 heavily pretreated patients, including 4 of 8 patients with anti–programmed cell death protein 1 (PD-1)-refractory disease. Toxicity was driven mainly by the preparative regimen rather than by unexpected on-target/off-tumor effects (92). On the basis of this signal, HPV16 E7-directed therapy has advanced into phase II clinical evaluation in metastatic HPV16-positive cancers (93). Together, these data position HPV16 E7 as the most active clinical TCR-T target currently reported in HPV-associated epithelial malignancies, whereas HPV16 E6 provided the earliest human feasibility signal.

More recent development has focused on broadening applicability beyond the original HPV16/HLA-A*02:01 setting. Preclinical work identified an HLA class II-restricted HPV18 E7-specific TCR with potent antitumor activity (94), and this receptor has now entered first-in-human phase I clinical testing in HPV18-positive solid tumors (95, 96). A second multicenter phase I study of the same HPV18-directed product is also ongoing, indicating that clinical development is beginning to expand across HPV subtype and HLA context, although mature efficacy data are not yet publicly available (96). Taken together, HPV-targeted TCR-T therapy remains one of the clearest early proof-of-concept settings in solid tumors, with the strongest current evidence coming from HPV16 E7-directed therapy and newer HPV18-directed programs extending the field into the next stage of clinical exploration.

### 3.8 EBV-associated malignancies

EBV-associated malignancies represent a biologically compelling yet still early setting for TCR-T therapy, with the strongest clinical evidence to date coming from EBV-positive nasopharyngeal carcinoma (NPC). In NPC, latency II restricts viral antigen expression primarily to Epstein–Barr nuclear antigen 1 (EBNA1), latent membrane protein 1 (LMP1), and latent membrane protein 2 (LMP2), thereby creating a limited but attractive repertoire for adoptive T-cell targeting (97).

Initial clinical proof of concept came from EBV-specific cytotoxic T-cell (CTL) therapy rather than engineered TCR-T. In relapsed NPC, allogeneic EBV-specific CTL infusion induced transient disease stabilization and enhanced LMP2-specific immunity (98), whereas subsequent studies of autologous EBV-specific T cells suggested more durable benefit, particularly in locoregional disease (99). In recurrent or metastatic NPC, however, efficacy has been less consistent. Although EBV-specific CTL therapy was feasible and well tolerated, the addition of lymphodepleting chemotherapy did not improve outcomes (100). A phase II study combining first-line gemcitabine/carboplatin with EBV-CTL reported a response rate of 71.4% (101). However, this signal was not confirmed in the randomized phase III VANCE trial (102), which showed no overall survival benefit despite favorable safety and successful centralized manufacturing. Taken together, these findings support the biological activity of EBV-directed adoptive cellular therapy in NPC, but durable and reproducible clinical benefit remains difficult to demonstrate in larger studies.

More recently, development has begun to shift from EBV-specific CTLs toward engineered receptor-based approaches. A notable transitional signal came from a patient with metastatic NPC who achieved a complete response to PD-1 blockade after prior EBV-specific adoptive T-cell therapy, suggesting potential synergy between virus-directed cellular therapy and checkpoint inhibition in selected cases (103). In parallel, engineered EBV-targeting TCR-T programs have entered early clinical testing. Preliminary first-in-human data were reported in 2023 for EBV-targeting TCR-T cells armored with secreted PD-1 blockade in EBV-positive NPC (104). Dedicated LMP2-specific TCR-T trials remain ongoing, whereas a cytokine-secreting EBV-specific TCR-T study has been completed but peer-reviewed efficacy and safety results have not yet been publicly reported (105, 106). Overall, EBV-associated NPC remains a promising but still early clinical setting for TCR-T therapy, in which EBV-specific CTLs provide the strongest current human evidence, whereas engineered TCR-T approaches are only beginning to define their therapeutic role.

Across tumor types, several common themes emerge. Actionable eligibility depends not only on HLA typing, but also on intact antigen-presentation competence, while barriers after target recognition vary considerably across TMEs. In this context, **Table 1** summarizes representative clinical studies together with selected ongoing or clinical-entry programs across solid tumors and highlights how target class, HLA restriction, safety, and clinical activity have shaped the current landscape of TCR-T development.

**Table 1:** Representative clinical studies and selected clinical-entry programs of TCR-engineered T-cell therapy in solid tumors.

Cancer type	Target antigen	HLA restriction	Epitope/peptide	Product	Trial ID/reference	Phase	N (treated)	Efficacy	Safety/take-home
Sarcoma	NY-ESO-1/LAGE-1a	HLA-A*02:01	SLLMWITQC	NY-ESO-1-directed TCR-T	Robbins et al., 2015	Pilot	18	ORR 61%	Acceptable safety in selected cohorts
Sarcoma	MAGE-A4	HLA-A*02:01P/*02:02P/*02:03P/*02:06P	GVYDGREHTV	afami-cel	NCT04044768	Phase II	52	ORR 37% overall; 39% in synovial sarcoma	Manageable CRS; cytopenias after lymphodepletion
Melanoma	MART-1	HLA-A*02:01	ELAGIGILTV	MART-1-directed TCR-T	Rohaan et al., 2022	Phase I/IIa	20	ORR 30%	Melanocyte-related on-target toxicity
Melanoma	gp100	HLA-A*02:01	KTWGQYWQV	gp100-directed TCR-T	Johnson et al., 2009	Phase I/II	16	ORR 19%	Lineage-related toxicity observed
Melanoma	NY-ESO-1	HLA-A*02:01	SLLMWITQC	NY-ESO-1-directed TCR-T	Robbins et al., 2015	Pilot	20	ORR 55%	Favorable relative to lineage-antigen programs
Melanoma	PRAME	HLA-A*02:01	SLLQHLIGL	IMA203	NCT03686124	Phase I	33	cORR 56% (18/32); DCR 91% (30/33)	Mostly low-grade CRS; limited ICANS
Melanoma	Individualized neoantigens	Individualized	Personalized neoantigen set	BNT221	NCT04625205	Phase I	9	Best response SD in 6/9; tumor reductions ≤20% in 4 patients	Well tolerated; no major unexpected toxicity
NSCLC	NY-ESO-1/LAGE-1a	HLA-A*02-eligible	SLLMWITQC	lete-cel	NCT02588612	Phase I	18	One durable response	Feasible safety profile
NSCLC/solid tumors	MAGE-A4	HLA-A*02-eligible	GVYDGREHTV	ADP-A2M4CD8	NCT04044859	Phase I	9 (NSCLC subset)	NSCLC subset: 1 PR, 2 SD/9	Manageable safety; severe CRS uncommon
PDAC	KRAS G12D	HLA-C*08:02	GADGVGKSA/GADGVGKSAL	KRAS G12D-directed TCR-T	Leidner et al., 2022	Case report	1	Objective regression of metastatic pancreatic cancer	Feasible in single-patient report
PDAC	KRAS G12V	HLA-A*11:01	VVGAVGVGK	KRAS G12V-specific TCR-T	NCT04146298	Phase I/II	5	1 CR lasting 5.5 months; 2 SD	Mainly hematologic toxicity; retreatment concerns
RCC	HERV-E	HLA-A*11:01	ATFLGSLTWK	HERV-E-directed TCR-T	NCT03354390	Phase I	15 infused	1 PR; ~29% SD ≥8 weeks	No major off-target toxicity reported
HBV-HCC	HBV surface antigen	HLA-matched	FLLTRILTI	HBV-specific mRNA TCR-T	NCT03899415; Meng et al., 2021	Phase I	8	One PR lasting 27.7 months	Well tolerated; transient-expression platform
HBV-HCC	HBsAg	HLA-A*02:01	FLLTRILTI	SCG101	NCT05339321	Phase I	6	Tumor shrinkage in 3/6 patients	Manageable hepatic toxicity; transient ALT elevation
HPV-associated epithelial cancers	HPV16 E6	HLA-A*02:01	TIHDIILECV	HPV16 E6-directed TCR-T	NCT02280811	Phase I/II	12	Two objective responses	No dose-limiting toxicity reported
HPV-associated epithelial cancers	HPV16 E7	HLA-A*02:01	YMLDLQPET	HPV16 E7-directed TCR-T	NCT02858310	Phase I	12	Objective responses in 6/12 patients	Mainly preparative-regimen-related toxicity
HPV-associated solid tumors	HPV18 E7	HLA-DRA/DRB1*09:01	HPV18 E7 84–98	HRYZ-T101	NCT05787535	Phase I (ongoing)	NR	Not yet reported	Safety not yet fully defined
HPV-associated solid tumors	HPV18 E7	HLA-DRA/DRB1*09:01	HPV18 E7 84–98	HRYZ-T101	NCT05952947	Phase I (ongoing)	NR	Not yet reported	Safety data immature
EBV-positive NPC	EBV antigen	Publicly not fully specified	Not publicly specified	EBV-targeting TCR-T with secreted PD-1 blockade	NCT04139057	Phase I/II (completed)	NR	Not yet reported	Early safety profile acceptable
EBV-positive NPC	LMP2	HLA-A*02:01/HLA-A*11:01/HLA-A*24:02 cohorts	Not publicly specified	LMP2-specific TCR-T	NCT03925896	Phase I (ongoing)	NR	Not yet reported	Safety not yet fully characterized
EBV-positive NPC	EBV antigen	Publicly not fully specified	Not publicly specified	EBV-specific cytokine-secreting TCR-T cells	NCT04509726	Phase I/II (completed)	NR	Not yet reported	Safety results not yet publicly reported
Solid tumors	TP53 R175H	HLA-A*02:01	HMTEVVRHC	NT-175	NCT05877599	Phase I (ongoing)	NR	Not yet reported	Safety data not yet mature
Solid tumors	KRAS G12D	HLA-C*08:02	GADGVGKSA/GADGVGKSAL	NT-112	NCT06218914	Phase I (ongoing)	NR	Not yet reported	Safety data not yet mature
Solid tumors	KRAS G12D	HLA-A*11:01*/*HLA-A*11:02	VVGADGVGK/VVVGADGVGK	AZD0240	NCT06218914	Phase I (ongoing)	NR	Not yet reported	Safety data not yet mature
Solid tumors	KRAS G12D	HLA-A*11:01	VVGADGVGK/VVVGADGVGK	KRAS G12D-reactive TCR-transduced lymphocytes (NCI)	NCT03745326	Phase I/II (ongoing)	NR	Not yet reported	Safety data not yet mature
Solid tumors	KRAS G12V	HLA-A*11:01	VVGAVGVGK	AFNT-211	NCT06043713	Phase I (ongoing)	NR	Not yet reported	Safety data not yet mature
mCRC	Individualized neoantigens	Individualized	Personalized neoantigen set	Personalized neoantigen-reactive TCR-transduced T cells	NCT03412877	Phase II	7	Early tumor regression in selected patients	Early safety acceptable

ORR, objective response rate; u/cORR, unconfirmed/confirmed objective response rate; cORR, confirmed objective response rate; CRS, cytokine release syndrome; PR, partial response; SD, stable disease; NR, not reported.

a, NCT06218914 is a KRAS TCR-T master protocol that includes distinct HLA-directed arms; NT-112 targets KRAS G12D in the context of HLA-C*08:02, whereas AZD0240 targets KRAS G12D in the context of HLA-A*11:01 or HLA-A*11:02.

b, For some EBV-directed studies, exact epitope information is not publicly specified.

## 4 Discussion and future directions

Across indications, proof-of-concept signals have emerged only when several conditions align: tractable targets, actionable patient eligibility, acceptable safety, preserved T-cell function after peptide–HLA recognition, and manufacturable products. Progress therefore depends on more than antigen selection alone. Safety, eligibility, post-recognition dysfunction, engineering strategy, and production feasibility interact to determine whether a TCR-T program can advance beyond early clinical activity.

### 4.1 Safety across target classes and treatment platforms

Across indications, the central safety lesson in TCR-T therapy is that peptide–HLA recognition is shaped not only by tumor specificity but also by low-level antigen display in normal tissues (6). Clinically, toxicity has followed two recurring patterns: on-target/off-tumor injury caused by intended recognition of physiologic antigen expression, and off-target toxicity caused by unanticipated peptide cross-reactivity. Landmark cases established both risks as design-level rather than disease-specific problems. In affinity-enhanced MAGE-A3 programs, fatal cardiogenic shock was linked to cross-recognition of a titin-derived peptide presented by HLA-A*01 (58). In a separate anti-MAGE-A3 setting, fatal neurotoxicity was associated with previously unrecognized MAGE-A12 expression in the brain (107). By contrast, CEA-directed TCR-T caused severe but reversible colitis, consistent with basal antigen expression in normal intestinal epithelium (108).

Beyond target choice, safety is also influenced by receptor configuration and engineering strategy. Potential mispairing between transgenic and endogenous TCR chains remains a theoretical and preclinical safety concern, because hybrid receptors with unpredictable specificity can be generated in experimental systems (65, 109). However, convincing clinical evidence that TCR mispairing has directly caused toxicity in humans remains lacking. This consideration nevertheless continues to motivate receptor-design strategies such as endogenous TCR disruption, optimized chain pairing, and targeted transgene insertion to reduce mispairing and normalize receptor expression (23, 110). A related lesson is that affinity optimization remains a trade-off: improved sensitivity to low-density peptide–HLA complexes may enhance tumor recognition, but excessive affinity can reduce discrimination between tumor and normal tissues and thereby increase the risk of cross-reactivity (111). Accordingly, preclinical safety evaluation increasingly relies on multilayered specificity assessment, including peptide-scanning approaches and cross-reactivity counterscreens across diverse cellular systems (112, 113). In parallel, toxicities such as CRS and ICANS should be addressed through predefined monitoring and management pathways, rather than being treated as downstream contingencies after efficacy signals emerge (114).

### 4.2 Beyond HLA typing: target prevalence, presentation competence, and actionable eligibility

A second cross-indication lesson is that actionable eligibility in TCR-T is determined by more than HLA matching alone. Rather, clinical deployability depends on the convergence of target prevalence, durable peptide–HLA presentation, and the frequency of the relevant HLA allele in the treatable population. This framework is illustrated by the contrast between established CTA-directed programs and more biologically specific targets. Even clinically validated targets may remain narrow in practice when they depend on restricted HLA backgrounds, as illustrated by afami-cel in MAGE-A4-positive synovial sarcoma, which still requires compatible HLA-A*02 alleles despite representing the most mature CTA-based regulatory precedent (54). Conversely, KRAS G12D-directed TCR-T exemplifies a setting in which the biology is compelling, yet real-world eligibility remains jointly constrained by mutation prevalence, peptide presentation, and allele frequency (21). HPV-directed TCR-T provides a parallel example: tumor specificity may be high, but actionable eligibility is still determined by biomarker-defined population size rather than by target biology alone (92).

**Figure 1:**
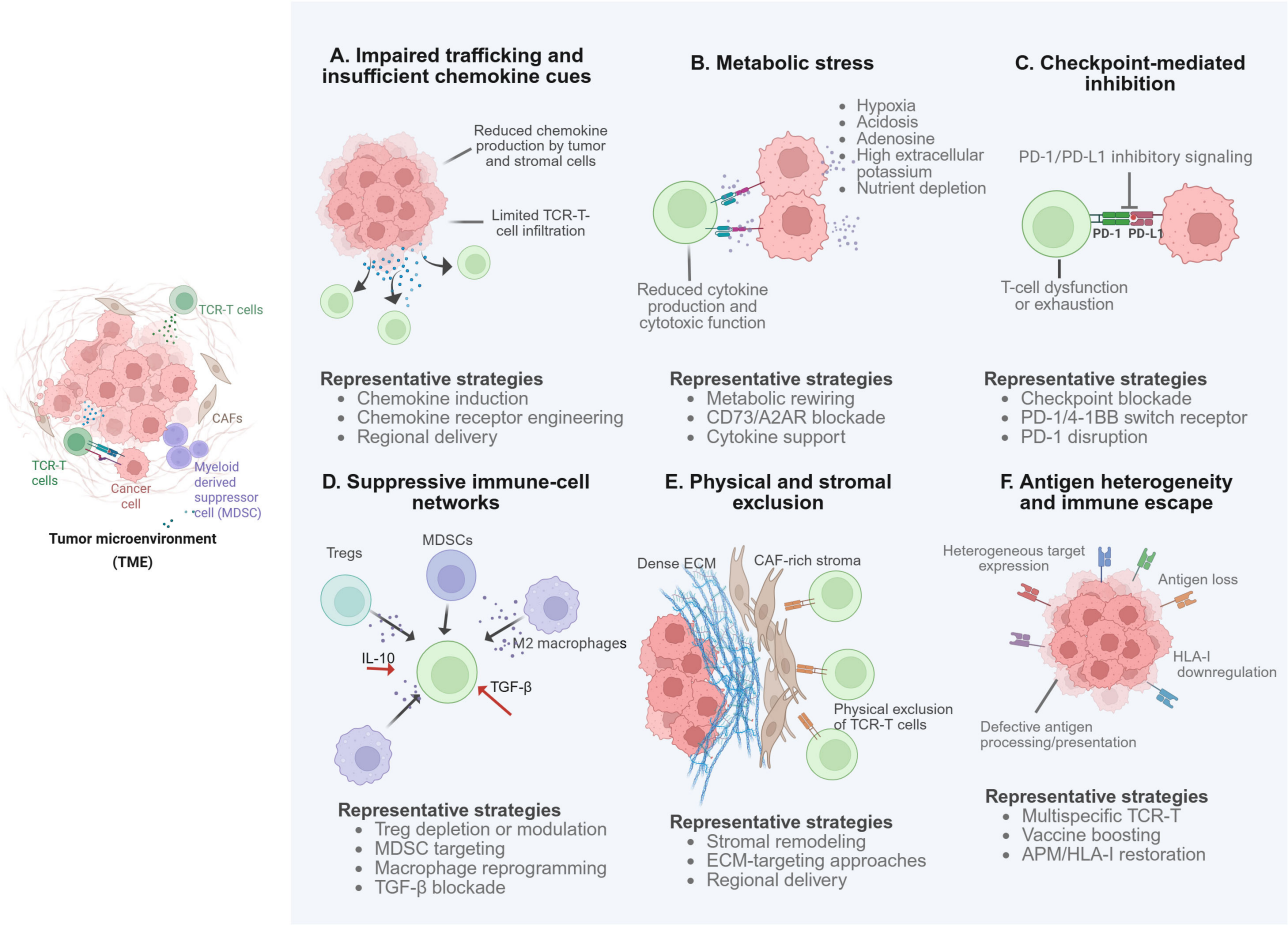
Major post-recognition barriers to TCR-T efficacy in the solid-tumor TME and representative mitigation strategies. Created in BioRender. Hu, W. (2026) https://BioRender.com/6kldwir.

A further recurrent constraint is antigen-presentation competence at the tumor level. Across target classes, apparent target positivity may still fail to translate into effective recognition if APM is defective or if HLA class I or β2-microglobulin expression is lost or suppressed (115). Clinically, these abnormalities may contribute to screening attrition, attenuated responses, and lesion-level heterogeneity despite nominal biomarker positivity. Accordingly, development programs are increasingly moving beyond HLA typing alone toward integrated biomarker frameworks that incorporate target expression, HLA class I and β2-microglobulin status, and, where feasible, more direct evidence that an actionable peptide–HLA complex is displayed on the tumor cell surface (116). Taken together, actionable eligibility in TCR-T is best understood as the convergence of target prevalence, stable presentation, and patient-specific immunogenetic context, rather than as HLA typing in isolation. However, actionable eligibility alone does not ensure effective tumor control after infusion. After target recognition, transferred T cells must still traffic to tumor sites, overcome stromal and metabolic constraints, resist suppressive signaling, and maintain function despite antigenic heterogeneity and immune escape (**Figure 1**).

The left inset provides an overview of the TME. Panels A–F summarize impaired trafficking and insufficient chemokine cues, metabolic stress, checkpoint-mediated inhibition, suppressive immune-cell networks, physical and stromal exclusion, and antigen heterogeneity or immune escape. Together, these barriers help explain why peptide–HLA recognition alone is often insufficient for durable tumor control in solid tumors.

### 4.3 Barriers after target recognition: trafficking, suppression, and persistence

Even when actionable eligibility is established and a relevant peptide–HLA target is present, durable tumor control is not assured. After infusion and initial target engagement, engineered T cells must still traffic to the tumor bed, penetrate stromal barriers, and preserve effector function within a suppressive TME (6). In solid tumors, dense extracellular matrix, stromal compartmentalization, and abnormal tissue architecture can restrict access to tumor nests even when antigen-specific T cells are detectable in the circulation (117). Once within the tumor, T cells may be further constrained by suppressive myeloid and regulatory populations, inhibitory cytokines such as TGF-β, and metabolically hostile conditions including hypoxia, nutrient deprivation, and adenosine accumulation (118, 119). These barriers help explain why evidence of target engagement does not necessarily translate into durable tumor regression in epithelial malignancies: effective therapy additionally requires successful trafficking, resistance to local suppression, and persistence of functional fitness over time.

### 4.4 Engineering strategies for dominant bottlenecks

These post-recognition barriers have directly shaped next-generation engineering strategies. To improve trafficking and local delivery, chemokine-receptor engineering seeks to better align transferred cells with tumor chemokine gradients (120). To counter suppressive signaling, platforms now incorporate dominant-negative TGF-β receptors (121), checkpoint switch receptors (122), and inverted cytokine receptors that either blunt inhibitory pathways or convert them into activating signals (123). Armored constructs secreting cytokines such as IL-12 or IL-18 aim to reinforce local immune activation and sustain intratumoral function (120). A further line of development targets metabolic resilience, particularly the adenosine axis, in an effort to preserve signaling and effector function in nutrient-poor and hypoxic tumor sites (124). Engineering strategies are most likely to succeed when matched to a defined bottleneck rather than applied as generic intensification measures, because each modification may improve intratumoral function at the cost of greater inflammatory risk or more complex toxicity control (121, 122).

### 4.5 Manufacturing, access, and product consistency

Manufacturing remains one of the central translational bottlenecks in solid-tumor TCR-T therapy. In autologous settings, vein-to-vein timelines may extend over several weeks, during which some patients deteriorate clinically or never reach infusion. This has increased interest in automation and closed-system manufacturing to reduce operator-dependent variability, improve sterility assurance, and support more reproducible production across sites (125, 126). In parallel, controlled genomic engineering—particularly targeted insertion into defined loci such as the T-cell receptor alpha constant (TRAC)—offers a route to standardize receptor expression and improve product consistency (127, 128). Manufacturing frameworks are also placing greater emphasis on critical quality attributes, including identity, viability, potency, purity, and vector-related metrics, as well as on defining how these variables relate to product function and safety (129). To broaden access, the field is also exploring allogeneic platforms (130). Renewable pluripotent stem-cell-derived immune-cell sources represent a parallel route to scalable off-the-shelf production (131, 132). *In vivo* engineering strategies may ultimately bypass parts of ex vivo manufacturing altogether (133).

## 5 Conclusion

TCR-T therapy has progressed from a mechanistically compelling concept to a clinically active and increasingly validated approach in selected biomarker-defined solid-tumor settings (52, 134). Its principal advantage lies in the ability to redirect T cells against intracellular tumor antigens presented as peptide–human leukocyte antigen (HLA) complexes, thereby extending targetability beyond cell-surface antigens. The clearest clinical value has so far emerged in biomarker-defined sarcoma and melanoma, where NY-ESO-1- and MAGE-A4-directed products have produced objective responses in prospective trials (50, 53), culminating in the regulatory approval of afami-cel for synovial sarcoma (54). By contrast, activity in most epithelial malignancies remains earlier in development, more heterogeneous, and generally less durable (49, 92). Current experience also makes clear that broader clinical applicability is limited not by a single factor, but by the combined constraints of actionable target prevalence, biomarker-defined eligibility, receptor specificity (27), and loss or instability of target presentation after therapy (135). The next phase of progress will therefore depend on three priorities: more rigorous target and epitope prioritization with systematic cross-reactivity de-risking (136); platform designs that better preserve antitumor function in solid-tumor TMEs (120, 121); and manufacturing advances that improve scalability, reproducibility, and product standardization (125, 126). Overall, TCR-T therapy now rests on a credible mechanistic and early clinical foundation in solid tumors, but broader clinical impact will depend on integrating precise targeting, barrier-aware product design, and robust manufacturing into durable and scalable treatment strategies.
