# Supplementary material for: Targeting solid tumors with TCR-T cells: mechanisms, progress, and challenges
Source: Front Oncol. 2026 May 7;16:1810903. doi: 10.3389/fonc.2026.1810903 (PMC13189840; doi:10.3389/fonc.2026.1810903)
Supplement: Supplementary file 1 [file Table1.docx]

Supplementary Table S1. Additional engineered TCR-T clinical studies in solid tumors.

| **Cancer type** | **Target antigen** | **HLA restriction** | **Epitope / peptide** | **Product** | **Trial ID / Reference** | **Phase** | **N (treated)** | **Efficacy** | **Safety / take-home** |
| --- | --- | --- | --- | --- | --- | --- | --- | --- | --- |
| Melanoma | PRAME | HLA-A*02:01 | PRAME-004 (SLLQHLIGL) | IMA203 (anzu-cel) | NCT06743126; JCO 2025 TPS2673 | Phase III | NR | NR | Ongoing randomized registration-stage trial |
| Melanoma / synovial sarcoma | PRAME | HLA-A*02:01 | PRAME-derived peptide; exact construct not publicly specified | IMA203 + mRNA-4203 | NCT06946225; JITC 2025 A671 | Phase I | NR | NR | First-in-human combination study; ongoing |
| NSCLC | MAGE-A10 | HLA-A*02 | MAGE-A10 254-262 (GLYDGMEHL) | ADP-A2M10 | NCT02592577; Blumenschein 2022 | Phase I | 11 | 1 PR and 4 SD among 11 treated patients | Acceptable safety; CRS reported, including 1 grade 4 event |
| Esophageal / GEJ cancers | MAGE-A4 | HLA-A*02 | MAGE-A4 230-239 (GVYDGREHTV) | ADP-A2M4CD8 (SURPASS-2) | NCT04752358 | Phase II | NR | NR | Registry-listed disease-specific expansion study |
| Mixed MAGE-A4+ tumors | MAGE-A4 | HLA-A*02 | MAGE-A4 230-239 (GVYDGREHTV) | ADP-A2M4CD8 +/- pembrolizumab | NCT05601752 | Phase II | NR | NR | Ongoing randomized expansion study |
| Advanced solid tumors | MAGE-A4/A8 | HLA-A*02:01 | MAGE-A4/A8-derived peptide(s); not publicly specified | IMA201 | NCT03247309 | Phase I | NR | NR | Early clinical development |
| Advanced solid tumors | MAGE-A1 | HLA-A*02:01 | MAGE-A1-derived peptide(s); not publicly specified | IMA202 | NCT03441100 | Phase I | NR | NR | Early clinical development |

Supplementary Table S1 (continued).

| **Cancer type** | **Target antigen** | **HLA restriction** | **Epitope / peptide** | **Product** | **Trial ID / Reference** | **Phase** | **N (treated)** | **Efficacy** | **Safety / take-home** |
| --- | --- | --- | --- | --- | --- | --- | --- | --- | --- |
| Advanced solid tumors | MAGE-A1 | HLA-A*02:01 | MAGE-A1-derived peptide(s); not publicly specified | TK-8001 (IMAG1NE) | NCT05430555 | Phase I/II | NR | NR | Recruiting first-in-human study |
| Epithelial cancers | KK-LC-1 | HLA-A*01:01 | KK-LC-1/CT83 52-60 (NTDNNLAVY) | KK-LC-1 TCR-T | NCT05035407; JCO 2022 TPS2678 | Phase I | NR | NR | Ongoing first-in-human study |
| Epithelial cancers | KK-LC-1 | Not publicly specified | KK-LC-1-derived peptide(s); exact sequence not publicly specified | KK-LC-1 TCR-T | NCT05483491 | Phase I | NR | NR | Parallel dose-escalation study |
| Metastatic solid tumors | MAGE-A3 | HLA-DP04:01/04:02 | MAGE-A3 243-258 (KKLLTQHFVQENYLEY) | anti-MAGE-A3-DP4 TCR PBL | NCT02111850 | Phase I/II | NR | NR | Registry-listed class II-restricted study |
| Melanoma / head and neck cancer | MAGE-C2 | HLA-A*02 | MAGE-C2 336-344 (ALKDVEERV) | MC2TCR | NCT04729543 | Phase I/II | NR | NR | Recruiting study |
| Metastatic cancers | MAGE-A3 | HLA-A*01 | MAGE-A3 168-176 (EVDPIGHLY) | anti-MAGE-A3 HLA-A*01-restricted TCR-T | NCT02153905 | Phase I/II | NR | NR | NCI treatment study; no mature efficacy publication identified |
| Advanced cancers | MAGE-A3/A6 | HLA-DPB1*04:01 | MAGE-A3/A6-derived DP4-restricted peptide(s); not publicly specified | KITE-718 | NCT03139370 | Phase I | NR | NR | Early study; no mature efficacy publication identified |

Supplementary Table S1 (continued).

| **Cancer type** | **Target antigen** | **HLA restriction** | **Epitope / peptide** | **Product** | **Trial ID / Reference** | **Phase** | **N (treated)** | **Efficacy** | **Safety / take-home** |
| --- | --- | --- | --- | --- | --- | --- | --- | --- | --- |
| Soft tissue sarcoma | NY-ESO-1 | HLA-A*02:01 | NY-ESO-1 157-165 (SLLMWITQC) | TAEST16001 | NCT04318964; Pan 2023 | Phase I | 12 | ORR 41.7%; median PFS 7.2 months | No treatment-related serious adverse events reported |
| Synovial sarcoma / melanoma | NY-ESO-1 | HLA-A2 | NY-ESO-1 157-165 (SLLMWITQC) | NY-ESO-1-specific TCR-T | NCT01343043; Robbins et al., J Clin Oncol, 2011 | Pilot | NR | Foundational pilot activity reported | Early pilot experience established feasibility |
| Bone / soft tissue sarcoma | NY-ESO-1 | Not publicly specified | NY-ESO-1-derived peptide; exact construct not publicly specified | NY-ESO-1-specific TCR-T | NCT03462316 | Phase I | NR | NR | single-arm phase I study; no mature efficacy publication identified |
| HBV-related HCC | HBV-derived antigen | HLA-A*02:01/24:02 | HBV-derived peptide(s); exact clinical epitope not publicly specified | SAFE-T-HBV | NCT04745403 | Phase I | NR | NR | Ongoing safety-focused study |
| Advanced solid tumors | KRAS G12V/G12D | Matching HLA-A subtype | KRAS G12V or G12D neoantigen / HLA-matched peptide(s) | Autologous TCR-T | NCT05438667; preprint-level update | Early Phase I | NR | NR | Early clinical development |
| Advanced solid tumors / PDAC-focused | KRAS G12V | HLA-A*11:01 | KRAS G12V neoantigen peptide | NW-301V | NCT06956261; ESMO 2025 Abstract 1514O (conference report) | Phase I | 14 | ORR 42.9% (6/14); DCR 78.6% (conference report) | conference-level data; registry status currently inconsistent with meeting report |
| Solid tumors | Personalized neoantigen | Personalized | Individualized neoantigen peptides | NeoTCR-P1 | NCT03970382 | Phase Ia/Ib | NR | NR | First-in-human gene-edited personalized TCR-T platform |

Supplementary Table S1 (continued).

| **Cancer type** | **Target antigen** | **HLA restriction** | **Epitope / peptide** | **Product** | **Trial ID / Reference** | **Phase** | **N (treated)** | **Efficacy** | **Safety / take-home** |
| --- | --- | --- | --- | --- | --- | --- | --- | --- | --- |
| Locally advanced or metastatic solid tumors | Individualized neoantigens | HLA- and antigen-matched | Cohort-/patient-specific peptide-HLA targets | Customized autologous TCR-T basket study | NCT05973487; T-Plex program | Phase I | NR | NR | Basket treatment study; no mature efficacy publication identified |
| HPV16+ solid tumors | HPV16 E7 | HLA-A*02:01 | HPV16 E7 11-19 (YMLDLQPET) | KITE-439 | NCT03912831 | Phase Ib | NR | NR | Registry-listed efficacy-oriented study |
| Metastatic HPV-associated cancers | HPV16 E7 | HLA-A*02:01 | HPV16 E7 11-19 (YMLDLQPET) | HPV16 E7 TCR-T cell immunotherapy | NCT05686226 | Phase II | NR | NR | Dedicated expansion study |
| Locoregionally advanced HPV-associated cancers | HPV16 E7 | HLA-A*02:01 | HPV16 E7 11-19 (YMLDLQPET) | HPV16 E7 TCR-T induction therapy | NCT05639972 | Phase I/II | NR | NR | Ongoing induction-strategy study |
| Advanced cervical / anal / head and neck cancers | HPV16 E7 | HLA-A*02:01 | HPV16 E7 11-19 (YMLDLQPET) | CRTE7A2-01 | NCT05122221 | Phase I | NR | NR | Early dose-escalation study |
| Advanced cervical / anal / head and neck cancers | HPV16 E7 | HLA-A*02:01 | HPV16 E7 11-19 (YMLDLQPET) | CRTE7A2-01 | NCT06358053 | Phase I | NR | NR | Dose-escalation/expansion study |
| Cervical carcinoma | HPV16 E6 | HLA-A*02 | HPV16 E6-derived peptide (TIHDIILECV) | HPV16 E6 TCR-T | NCT05357027 | Phase I/II | NR | NR | Recruiting study |

Supplementary Table S1 includes additional engineered TCR-T studies not prioritized for the main table because they are early-phase, mixed-cohort, registry-based, conference-level, personalized, or lacked mature disease-specific efficacy readouts at the time of writing. NR indicates not reported in a stable public efficacy publication. Abbreviations: ORR, objective response rate; PFS, progression-free survival; DCR, disease control rate; CRS, cytokine release syndrome; DLT, dose-limiting toxicity; ICANS, immune effector cell-associated neurotoxicity syndrome; GEJ, gastroesophageal junction; SD, stable disease; PR, partial response.
